# Genetic variations in the serotonergic system contribute to amygdala volume in humans

**DOI:** 10.3389/fnana.2015.00129

**Published:** 2015-10-09

**Authors:** Jin Li, Chunhui Chen, Karen Wu, Mingxia Zhang, Bi Zhu, Chuansheng Chen, Robert K. Moyzis, Qi Dong

**Affiliations:** ^1^State Key Laboratory of Cognitive Neuroscience and Learning, IDG/McGovern Institute for Brain Research, Beijing Normal UniversityBeijing, China; ^2^Brainnetome Center, Institute of Automation, Chinese Academy of SciencesBeijing, China; ^3^National Laboratory of Pattern Recognition, Institute of Automation, Chinese Academy of SciencesBeijing, China; ^4^Center for Collaboration and Innovation in Brain and Learning Sciences, Beijing Normal UniversityBeijing, China; ^5^Department of Psychology and Social Behavior, University of California, IrvineIrvine, CA, USA; ^6^Key Laboratory of Behavioral Science, Institute of Psychology, Chinese Academy of SciencesBeijing, China; ^7^Department of Biological Chemistry, University of California, IrvineIrvine, CA, USA; ^8^Institute of Genomics and Bioinformatics, University of California, IrvineIrvine, CA, USA

**Keywords:** serotonin, gene, amygdala, brain structure, missing heritability

## Abstract

The amygdala plays a critical role in emotion processing and psychiatric disorders associated with emotion dysfunction. Accumulating evidence suggests that amygdala structure is modulated by serotonin-related genes. However, there is a gap between the small contributions of single loci (less than 1%) and the reported 63–65% heritability of amygdala structure. To understand the “missing heritability,” we systematically explored the contribution of serotonin genes on amygdala structure at the gene set level. The present study of 417 healthy Chinese volunteers examined 129 representative polymorphisms in genes from multiple biological mechanisms in the regulation of serotonin neurotransmission. A system-level approach using multiple regression analyses identified that nine SNPs collectively accounted for approximately 8% of the variance in amygdala volume. Permutation analyses showed that the probability of obtaining these findings by chance was low (*p* = 0.043, permuted for 1000 times). Findings showed that serotonin genes contribute moderately to individual differences in amygdala volume in a healthy Chinese sample. These results indicate that the system-level approach can help us to understand the genetic basis of a complex trait such as amygdala structure.

## Introduction

The amygdala, an almond-shaped brain structure which resides in the medial temporal lobe of the brain (Whalen and Phelps, 2009), is key in emotion processing (Sergerie et al., 2008). Lesion studies suggest that the amygdala plays a central role in the perception of emotional stimuli (Campanella et al., 2014), and fMRI studies show that the amygdala activates in response to emotional stimuli (Habel et al., 2007). Accordingly, the volume of the amygdala is a widely used index of emotional processing in both animal and human studies (Yang et al., 2008; Hartley et al., 2011). Rodent studies have found an association between amygdala volume and variation in emotion learning (Yang et al., 2008), while accumulating clinical studies have discovered volume abnormalities of the amygdala in patients with depression (Rubinow et al., 2014), bipolar disorder (Lisy et al., 2011), and borderline personality disorder (Ruocco et al., 2012). For instance, patients with personality disorder have a 13% smaller amygdala than healthy controls (Ruocco et al., 2012). Given these findings, amygdala volume could be a promising endophenotype in regards to emotional behavior and related psychiatric diseases.

Moderate to high heritability for amygdala volume (Hulshoff Pol et al., 2006; Kremen et al., 2010) suggest a significant genetic basis for this trait. The amygdala is densely innervated by serotonergic fibers (Bauman and Amaral, 2005), and the influence of synaptic serotonin on amygdala responsiveness has been identified (Fisher et al., 2006; Rhodes et al., 2007). Accumulating imaging genetics studies have linked individual differences in amygdala volume to genes affecting serotonergic signal. These genes encode proteins involved in serotonin synthesis (Inoue et al., 2010), reuptake (Frodl et al., 2008; Stjepanovic et al., 2013), metabolic degradation (Meyer-Lindenberg et al., 2006), and receptors (Zetzsche et al., 2008). However, the variance of the amygdala structure explained by a single genetic locus is small, no more than 1% (Hibar et al., 2015), far less than the reported 63–66% heritability (Kremen et al., 2010) from twin studies. This gap might be explained as the amygdala size, like many complex quantitative traits, is influenced by multiple genes, each with a small effect. Therefore, a gene set based model is needed to assess the additive effects of a group of functionally related genes that mediate a particular biological process (i.e., serotonin functioning) and potential interactions.

In the present study, we applied the system-level approach developed by our research group (Chen et al., 2015) to evaluate the overall contribution of the serotonin system genes on individual differences in amygdala morphology. A large sample of 417 Han Chinese adults was recruited, and 129 polymorphic loci within the serotonin system were genotyped to cover a substantial portion (by LD) of the common variations in serotonin system genes, including biosynthesis, vesicular release, active reuptake, metabolic degradation, and presynaptic and postsynaptic receptors. We hypothesized that the genes along the specified pathway would contribute greatly to variation in amygdala volume.

## Materials and Methods

### Participants

Our 417 participants (mean age 20.4 years, *SD* = 0.9; 179 males and 238 females) were a subset of a larger study of 480 healthy Chinese college students (mean age = 19.9 years, *SD* = 0.9; 208 males and 272 females) from Beijing Normal University, Beijing, China (Li et al., 2011), for whom structural imaging data was available. All participants were Han Chinese and were free of neurological and psychiatric disorders. This study was approved by the IRB of the State Key Laboratory of Cognitive Neuroscience and Learning at Beijing Normal University, China. All experiments were performed in accordance with approved guidelines and regulations. Written informed consent was obtained from each participant.

### Gene Selection

We selected genes using the serotonin pathway defined in the Kyoto Encyclopedia of Genes and Genomes database, a collection of pathway maps widely used in gene-set analysis. Genes in the following four serotonin subsystems were selected: (1) the serotonin synthesis subsystem, which converts hydroxylation (by TPH) to 5-HT: *TPH1*, *TPH2*; (2) the degradation subsystem, which directly breaks down released 5-HT at the synapse into inactive metabolites (*MAOA*, *MAOB)*; (3) the transportation subsystem, which pumps serotonin from synaptic spaces into presynaptic neurons [*SLC6A4*(also known as *5-HTT*)] or integrates the membrane of intracellular vesicles of presynaptic neurons and transported monoamines into the synaptic vesicles [*SLC18A1*(also known as *VMAT1*), *SLC18A2*(also known as *VMAT2*)]; (4) the serotonin receptor subsystem (*HTR1A, HTR1B, HTR1D, HTR1F, HTR2A, HTR2B, HTR2C, HTR3A, HTR3B, HTR3C, HTR3D, HTR3E, HTR4, HTR5A, HTR5B, HTR6, HTR7*). Together, the selected genes represent all major genes involved in the four serotonin subsystems in humans (Chen et al., 2015). Several tag SNPs (tSNPs) defined by the HapMap project^1^ [Phase 3] (Frazer et al., 2007) were selected to sample the genetic diversity of these genes. Details of these genes and the selected loci (129 polymorphisms, including 127 SNPs and 2 VNTR polymorphisms) are shown in Supplementary Table S1.

### Genotyping Techniques

Genotyping was conducted as previously described (Li et al., 2011). Briefly, 4 ml venous blood sample was collected from each subject, and then genomic DNA was extracted according to standard methods. SNPs were genotyped using the Illumina GoldenGate Genotyping protocol (see Illumina GoldenGate Assay Protocol for details^2^). In addition, two genetic markers (*5-HTTLPR*, *MAOA* VNTR) were ascertained by standard PCR procedures (Chen et al., 2015).

### Gene Data Preprocessing

Quality control of the genetic data was carried out based on the larger sample of 480 participants. Two subjects met the criteria of over 10% null genotyping, and were thus excluded from subsequent analyses. Of the 60228 genotypes (126 SNPs by 478 subjects), 120 genotypes (0.2%) were excluded because of low GenCall (<0.25). If any SNP had fewer than 10 (2%) heterozygotes or minor homozygotes, these two genotype groups were combined. If the combined group still had fewer than 10 subjects, that SNP was excluded from further analysis. We found that five SNPS showed significant Hardy–Weinberg disequilibrium (*p* < 0.01) based on a *df* of 1 (for SNPs located on X chromosome, only females were included in HWE calculation since males have only one X chromosome). However, these SNPs were retained because the HW disequilibrium here did not seem to result from genotyping error but rather reflected the characteristics of college students due to social selection (i.e., overrepresentation of alleles linked to school achievement and motivation; Chen et al., 2013). Because both tag SNPs and additional SNPs in regions detected in recent selection (Hawks et al., 2007) were selected in the current study, there was high LD among some SNPs. Thirty SNPs were excluded from multiple regression analysis because of their high LD with adjacent SNPs [*r*^2^ > 0.8, Plink calculated (Purcell et al., 2007)]. Genetic relatedness among subjects was checked following Anderson et al. (2010) protocol by Plink. All genotyped unrelated autosome SNPs (*n* = 240, *r*^2^ < 0.8) were used and the threshold was set at 0.95 (personal communication with Dr. Anderson and Dr. Zondervan). No pair of subjects showed high relatedness (all PI_HAT smaller than 0.5).

Four-hundred and seventy-eight subjects (99.6%) and 99 polymorphisms (77%) passed all of the aforementioned quality control procedures. Of these 478 subjects, 417 had structural imaging data and were thus included in the subsequent analyses of the 99 polymorphisms. The information for all 129 loci (127 SNPs and 2 VNTRs) is shown in Supplementary Table S1, including location (rs number, chromosome, position), gene, serotonin subsystem, allele polymorphism and frequency, HWE, LD, and whether they were included in the main analyses.

### MRI Data Collection

MRI scans were performed in a 3.0T Siemens Magnetom Trio scanner equipped with a standard head coil at Beijing Normal University Brain Imaging Center. Structural MRI data were acquired with the T1-weighted MPRAGE pulse sequence (TE = 3.75 ms, TR = 2,530 ms, flip angle = 7°, FOV = 256 mm × 256 mm, voxel size = 1 mm × 1 mm × 1.33 mm, number of partitions = 128).

### MRI Data Processing

Cortical surface reconstruction and volumetric segmentation were performed with the FreeSurfer software^3^ (Version 4.5.0). Each subject’s average T1-weighted image was segmented into gray matter volumes for seven subcortical regions relying upon variations in voxel signal intensities, probabilistic atlas location and local spatial relationships between the structures (Fischl et al., 2002). Quality control of scan images and segmentation was assured by visual inspection of the whole cortex of each subject, and any inaccuracies in Talairach-transformation, skull stripping and segmentation were manually corrected and re-inspected. High correlations between the automatic measures and manual measures *in vivo* and *ex vivo* have been demonstrated (Desikan et al., 2006). The amygdala volumes obtained from the Freesurfer procedure have been reported to be significantly correlated with manual parcellation (Stjepanovic et al., 2013).

Volumes of bilateral amygdala were retracted from the standard output of the FreeSurfer analysis. Then the mean bilateral amygdala volume was calculated. A preliminary analysis showed that both gender and ICV were significantly associated with amygdala size (for gender, *F*(1,415) = 48.06, *p* = 1.59 × 10^-11^; for ICV, *F*(1,415) = 114.23, *p* = 1.03 × 10^-23^). Therefore, to control for the confounding effects of gender and ICV, a regression analysis was conducted, with gender and ICV as independent variables, and amygdala size as the dependent variable. The residual for each subject was normally distributed (skewness = 0.456, kurtosis = 0.747, *Kolmogorov–Smirnov* test = 0.039, *p* = 0.136) and was used as an index of amygdala volume in subsequent association analyses. To avoid the possible confounding effect of emotion state on amygdala structure, the associations between Beck Anxiety Inventory (BAI) and Beck Depression Inventory (BDI) scores with amygdala size were tested separately. The associations did not reach significance [for BAI, *F*(1,415) = 0.74, *p* = 0.39; for BDI, *F*(1,415) = 2.67, *p* = 0.10]. Therefore, the BAI and BDI scores were not considered in subsequent analyses.

### Statistical Analysis

The statistical procedure was comprised of three major analyses. First, analysis of variance (ANOVA) was conducted for each of these loci to detect variants which met the inclusion criterion (*p* < 0.05, uncorrected, to control for Type II error). Second, these loci were then entered into a regression model to estimate their overall contribution to amygdala size. In the regression model, all loci with significant main effects based on the ANOVA results were included with a forward stepwise method. In this step, all SNPs were coded in a linear way, i.e., the major homozygote, heterozygote, and minor homozygote were coded into 1, 2, and 3, respectively (SNPs on X chromosome were coded as 1 and 3 for major and minor allele homozygote, and also 3 for female heterozygotes). In addition, the *MAOA* VNTR was coded as 1 for the 3 repeat and 3 for the 4 repeat in males and 1 for 3 repeat homozygotes and 3 for others in females. Finally, the regression model was verified by permutation. Permutation tests were done 1000 times by shuﬄing amygdala volume data across subjects. In each iteration, selection of significant snps from the ANOVA tests, regression model estimation with a forward stepwise method, and *r*^2^ calculation were carried out on the shuﬄed data. The probability of getting a larger *r*^2^ in the shuﬄed data than in the real data was defined as *p*-value of the model.

## Results

The mean bilateral amygdala volume across subjects was 1700.5 mm^3^ (*SD* = 177.7 mm^3^). ANOVA was used to screen the 99 loci that passed quality control procedures for associations with the amygdala size. Nine SNPs showed main effects on amygdala volume with uncorrected *p* < 0.05. Specifically, individuals who were major allele homozygotes for rs7997012 (*HTR2A*), rs7984966 (*HTR2A*), rs939334 (*HTR3D*), rs10917509 (*HTR6*), or rs363226 (*SLC18A2*), or minor allele homozygotes for rs1487275 (*TPH2*), rs6792482 (*HTR3D*), rs11676829 (*HTR5B*), or rs12249377 (*HTR7*), tended to have larger amygdala size than the remaining groups. (For details, see **Table 1**, and online Supplementary Table S2 ).

**Table 1:** Means and standard deviations of amygdala volume for each polymorphism in nine significant SNPs, and *post hoc* comparisons of each locus.

Subsystem	Gene	SNP	Maj	Mean	*SD*	*N*	Het	Mean	*SD*	*N*	Min	Mean	*SD*	*N*	*F*	*p*	*Post hoc* (*p* < 0.05)
Synthesis	*TPH2*	rs1487275	AA	1723.66	14.29	174	AC	1709.73	181.82	188	CC	1775.90	25.41	55	3.68	0.03	AA, AC < CC
Transport	*SLC18A2*	rs363226	CC	1735.34	186.30	349	CG	1667.48	194.85	68	GG				8.99	0.00	
	*HTR2A*	rs7997012	GG	1718.46	196.29	230	AG	1713.51	170.93	150	AA	1804.02	200.47	37	3.87	0.02	GG, AG < AA
	*HTR2A*	rs7984966	AA	1731.00	190.66	381	AG	1653.04	157.77	36	GG				6.48	0.01	
Receptor	*HTR3D*	rs939334	AA	1739.33	180.99	216	AG	1722.04	200.46	161	GG	1655.10	172.98	39	3.54	0.03	AA > GG
	*HTR3D*	rs6792482	GG	1689.61	180.70	106	AG	1729.74	184.16	209	AA	1749.08	204.04	102	4.38	0.01	GG < AG, AA
	*HTR5B*	rs11676829	AA	1715.14	186.37	310	AG	1737.48	186.99	99	GG	1914.46	236.80	8	3.71	0.03	AG < GG
	*HTR6*	rs10917509	AA	1738.57	190.66	269	AG				GG	1698.28	184.18	148	5.38	0.02	
	*HTR7*	rs12249377	CC	1735.40	196.98	298	AC	1690.54	154.53	109	AA	1760.23	259.87	10	3.87	0.02	AC < AA, CC

*(a) Empty cells indicate that no such genotypes were found in our sample or that *post hoc* comparisons were not run because there were only 2 groups for this locus. Maj, Major allele; Het, Heterozygote; Min, Minor allele*.

*(b) A preliminary regression analysis was carried out to control for the influence of gender and ICV on average amygdala size. The F, *p*-value and the *post hoc* results in the table came from ANOVA on the residual of this regression*.

These nine SNPs were added to a regression model using the forward stepwise procedure to estimate their overall contribution to amygdala size. five of them made significant and unique contributions to the final model, while the other four SNPs were not included because of collinearity with other SNPs (see **Table 2**). The regression model accounted for 8.2% (6.8% adjusted) of the variance in amygdala size [*F*(5,417) = 7.33, *p* = 1.3 × 10^-6^]. The confidence interval of *R*^2^ estimated by bootstrap for 1000 times was 0.04–0.16, and that for adjusted *R*^2^ was 0.03–0.15.

**Table 2:** Regression model for amygdala volume with genetic data.

Regressor	Gene	*B*	*T*	*p*
rs10917509	HTR6	-21.16	-2.56	0.01
rs11676829	HTR5B	48.88	3.00	0.00
rs6792482	HTR3D	30.62	2.73	0.01
rs363226	SLC18A2(VMAT2)	-58.70	-2.73	0.01
rs7984966	HTR2A	-65.77	-2.30	0.02

*‘Gene’ is the corresponding gene for each SNP; ‘*B*’ is the regression coefficient, ‘*T*’ and ‘*p*’ are *t*-test results. Effects of gender and ICV are controlled prior to this analysis*.

**FIGURE 1:**
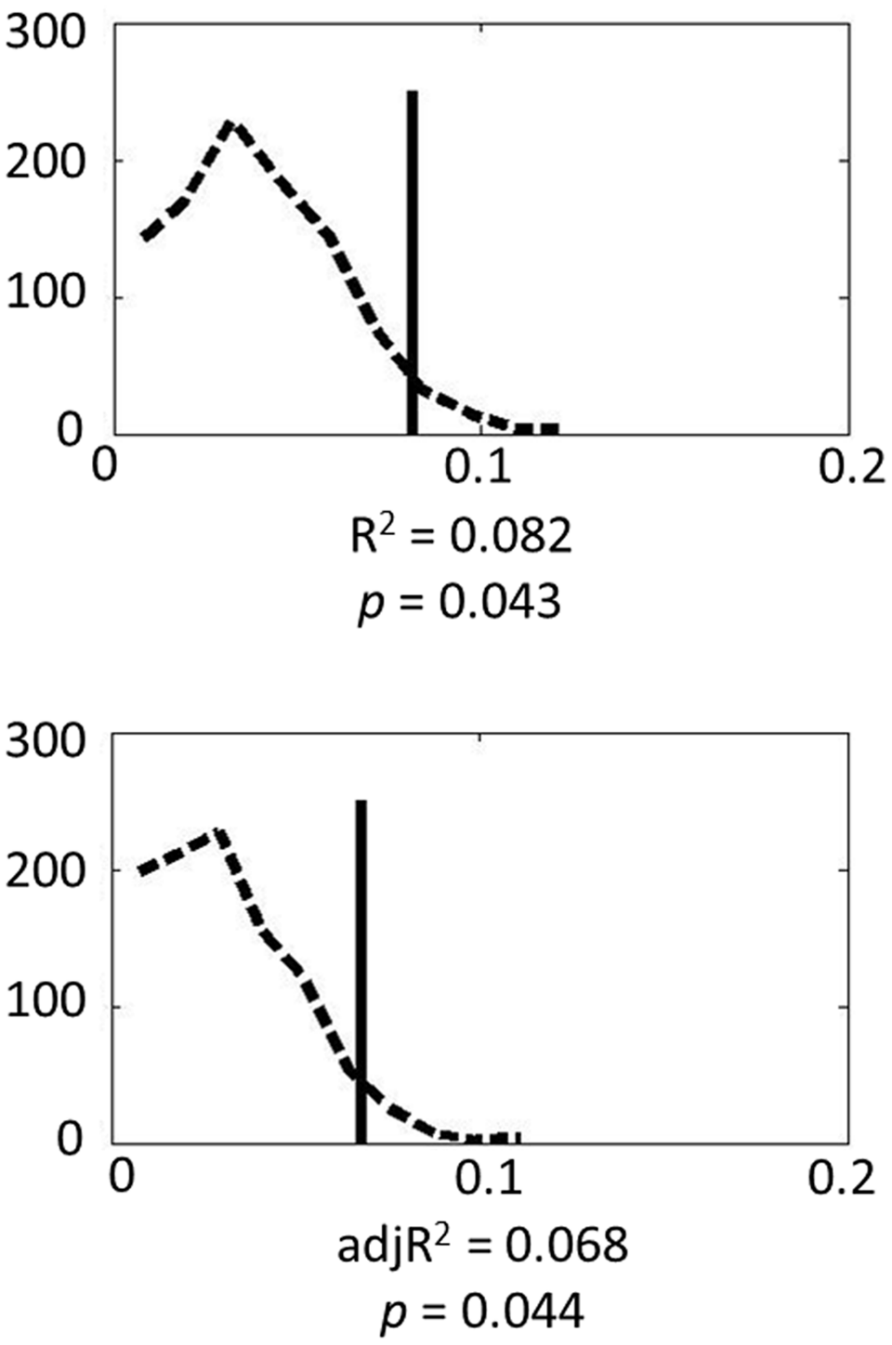
**Permutation results for the genetic model: the dashed line represents the empirical distribution of *R*^2^ obtained from the randomized data, and the solid vertical line represents *R*^2^ obtained from the actual data**.

Finally Monte Carlo permutation analyses were carried out to test the model. **Figure 1** shows the permutation results. Based on 1000 permutation tests, the probability of attaining the *R*^2^ or adjusted *R*^2^ found in the model reached significance (*p* = 0.043 and 0.044, respectively). These results indicate that genes in the serotonin system contribute substantially to individual variance in amygdala volume.

## Discussion

The current study combined the advantages of both the candidate gene approach and dense genotyping technology. Our theory-driven method detected a group of biological relevant genes based on prior knowledge and thus avoided the heavy comparison correction necessary in GWAS. However, unlike candidate gene studies on single genes, our system level approach took into account the polygenic nature of amygdala structure. Given the innervation of serotonergic fibers in the amygdala (Bauman and Amaral, 2005) and the role of serotonergic genes on amygdala structure (Meyer-Lindenberg et al., 2006; Frodl et al., 2008; Zetzsche et al., 2008), we used dense gene chips to cover all tag SNPs of the serotonergic biological pathway in order to test the additive effect of potential genes. Results suggest that such a system level approach could bridge the gap between the small contributions of single genes and the considerable heritability of amygdala volume revealed by twin studies. Specifically, serotonergic genes collectively accounted for 8.2% of variance in amygdala volume. Although associations between these specific SNPs and amygdala structure have not been reported before, direct and indirect evidence have linked these genes to amygdala structure and amygdala-related psychological disorders. In the following paragraphs, we discuss each of these genes.

The *TPH2* gene encodes TPH protein which is involved in the rate-limiting biosynthesis of serotonin. Postmortem studies have revealed the expression of *TPH2* mRNA in the amygdala (Zill et al., 2007). Raphe neurons of *Tph2* knockout mice were completely devoid of 5-HT, indicating that brain 5-HT synthesis across the lifespan is exclusively maintained by TPH2 (Gutknecht et al., 2009). Previous imaging genetic studies also found associations between several *TPH* SNPs and the amygdala, such as rs4570625 with the structure (Inoue et al., 2010) and function (Furmark et al., 2009) of the amygdala, and rs17110563 with bipolar disorder (Cichon et al., 2008). Thus far, however, no study has linked rs1487275 genotype to any emotion-related behavior or psychiatric disease, although its effect on amygdala structure was identified in the current study. Therefore, future studies should explore such potential associations in healthy or clinical samples.

*SLC18A2* encodes VMAT2 that transports free serotonin from cellular cytosol into synaptic vesicles (Eiden et al., 2004). Rodent studies have reported early expression of *VMAT2* in amygdala (Lebrand et al., 1998) and that mice lacking one copy of the *VMAT2* gene develop with significantly reduced serotonin (Fon et al., 1997). Convergent studies have linked *VMAT2* gene to brain development and amygdala-related psychiatric diseases. For instance, an increase in cell death in the superficial layers of the cingulate and retrosplenial cortices during early postnatal life in *Vmat2* knockout mice (Stankovski et al., 2007) and a delayed maturation of the upper cortical layers in the *Vmat2*(sert-cre) and *Tph2*(-/-) mice (Narboux-Nême et al., 2013) were reported. Moreover, *VMAT2* heterozygous mice exhibit ‘depression-like’ phenotype (Fukui et al., 2007). In human studies, patients with bipolar disorder showed higher binding of VMAT2 (Zubieta et al., 2001), patients with major depression also showed elevated VMAT2 density (Zucker et al., 2002) and structural change of VMAT2 (Zalsman et al., 2011) in platelets. Our finding of an association between *SLC18A2* variation and the structure of the amygdala seems in accordance with the above previous results.

*HTR2A*, *HTR3D*, *HTR5B*, *HTR6*, and *HTR7* encode different serotonin receptors. Studies indicated the expression of *HTR2A* (McDonald and Mascagni, 2007), *HTR3* (Morales et al., 1998), *HTR6* (Marazziti et al., 2012) in the amygdala. Their effects on amygdala structure might be partly accounted for by the distribution of serotonin receptors in the amygdala, and the modulation effect of serotonin receptors on different developmental processes (Gaspar et al., 2003), such as neurogenesis, apoptosis, axon branching, and dendritogenesis. Accumulating pharmacological studies have linked these receptors to the function of the amygdala and related psychiatric diseases. For example, HTR2 agonist has been found to increase neuronal firing of the amygdala (Stein et al., 2000) and to increase anxiety-like behavior (Pockros-Burgess et al., 2014); HTR2A antagonist can have an antidepressant-like effect (Quesseveur et al., 2013); HTR3 agonist attenuates antidepressants’ effect (Nakagawa et al., 1998), whereas HTR3 antagonist as well as HTR6 and HTR7 have an antidepressant effect (Wesolowska and Nikiforuk, 2007; Mnie-Filali et al., 2011; Gupta et al., 2014). A recent study also showed that social isolation stress could result in up-regulation of HTR5B, suggesting a close link between HTR5B and emotion and its neural substrates such as the amygdala (Maekawa et al., 2010). In addition to pharmacological studies, at least one molecular genetic study found a significant association between rs7997012 variation (*HTR2A*) and the therapeutic response to antidepressant treatments in major depression patients (Lin et al., 2014). In sum, previous studies have consistently shown that the above serotonin receptors play a major role in mood disorders, which are likely related to amygdala dysfunction. Moreover, *HTR6* was indicated to mediate brain development in MAOA-deficient mouse embryos (Wang et al., 2014) and *HTR7* signaling was reported to regulate neuronal morphology (Kobe et al., 2012). These documented genetic effects on neural development may have a permanent impact on the size of the amygdala. Given that the formation of serotonergic neurons and fiber distribution are not impaired (Gutknecht et al., 2008) inTph2 knockout mice (that are completely deficient in brain serotonin synthesis), we inferred that the effect of these genes on amygdala size was not through serotonergic neurons but through serotonin level and its effect on brain development. In the developing nervous system, an excess of serotonin affects interneuron migration (Riccio et al., 2009) and neocortical pyramidal neuron migration (Riccio et al., 2011). High levels of serotonin are also suggested to have neuroprotective effects on cortical neurons (Stankovski et al., 2007), while lack of brain serotonin is suggested to affect postnatal development and serotonergic neuronal circuitry formation (Migliarini et al., 2013). In summary, the association that we found between serotonergic genes and amygdala structure might result from the developmental role of serotonin.

Further research is required to support our findings for several reasons. First, some serotonergic genes, which have been found in several studies to impact amygdala structure, were not found to impact amygdala morphology in the present study (e.g., *HTR1A*). One possible explanation is that *HTR1A* may be important in amygdala structure and function in Caucasians (for whom most previous studies were on), but not Chinese. We were not surprised at the negative result in our Han Chinese sample, as several studies have reported that the same genetic variation can result in divergent psychological outcomes, depending on the population (Long et al., 2013; Wang et al., 2013). For example, a recent study genotyped the *HTR1A* polymorphism in European Americans and Koreans, and reported a significant interaction between *HTR1A* genotype and culture in the locus of attention (Kim et al., 2010). Moreover, the association between the *HTR1A* gene polymorphism (rs6295) and bipolar disorder in the Caucasian sample (Sullivan et al., 2009) was not found in the Korean population (Kim et al., 2014). Therefore, our sample of pure Han Chinese helps to prevent the confounding effect of population, but we should be cautious as it also limits the generalization of our results to other populations. Another possible explanation is that we may have missed the causative SNPs by using only tag SNPs to sample the genetic diversity of these genes. Further studies are required to test this association in other populations and to genotype more SNPs.

Second, in the current study, we focused on the role of serotonergic genes in healthy young adults to avoid the confounding effects of neurological diseases and age (Filippini et al., 2009). However, it should be noted that both developmental mechanisms and adult chronic disease may affect amygdala size, but through different mechanisms. Studies involving larger sample sizes and older adults (to explore possible effects of aging) and subjects with chronic disease are needed. Third, considering that amygdala dysfunction accompanying structural abnormality also underlies emotion related psychiatric disorders (Ubl et al., 2015) and reflects the effect of serotonergic genes (Hariri et al., 2002), further studies are needed to test the functional indices of the amygdala. Also, studies on amygdala subregions using higher resolution images could provide more information regarding the effects of serotonergic genes on the amygdala. Fourth, the current study could not identify the specific serotonin receptor(s) that transduced the effects. More direct biological evidence is required in further studies to elucidate the relationship between the serotonergic receptors and the downstream amygdala structural change.

Last but not the least, all associated SNPs in our study were located in non-coding regions. This result is consistent with the view that non-coding regions which were once labeled as “junk DNA” actually may play important functional roles (Birney et al., 2007). Some studies indicate that intron variants are involved in gene expression (Zhang et al., 2007) or mRNA secondary structure formation (Nackley et al., 2006). To explore the specific roles of the SNPs screened in our study, more systematic studies using animal models and other techniques (e.g., optogenetics) are required. Third, although genes from the serotonin system accounted for 8.2% of the variance in amygdala volume, there is still much more “missing heritability” (8.2% vs. 63–65% heritability) to be accounted for. Future studies regarding amygdala structure should incorporate other genetic systems, environmental factors, genetic epistasis, and gene-environmental interactions.

## Conclusion

Our system-level approach indicated that several genes within the serotonin system had small effects on amygdala structure, and these genes together accounted for a sizable portion of the missing heritability of amygdala volume. The system-level analysis may enhance our understanding of the genetic basis of human amygdala structure and amygdala-related emotional behaviors and psychiatric diseases.

## Conflict of Interest Statement

The authors declare that the research was conducted in the absence of any commercial or financial relationships that could be construed as a potential conflict of interest.

## Footnotes

1 www.hapmap.org

2 www.southgene.com.cn

3 http://surfer.nmr.mgh.harvard.edu

## ABBREVIATIONS

5-HT: 5-hydroxytryptamine
5-HTT: serotonin transporter
5-HTTLPR: serotonin transporter gene linked polymorphic region
GWAS: Genome-Wide Association Study
HTR: serotonin receptor
ICV: intracranial volume
LD: linkage disequilibrium
MAOA: Monoamine oxidase A
MAOB: Monoamine oxidase B
SNP: Single Nucleotide Polymorphism
TPH: tryptophan hydroxylase
VMAT: Vesicular monoamine transporter
VNTR: variable number tandem repeat
