# Supplementary material for: Genetic variations in the serotonergic system contribute to amygdala volume in humans
Source: Front Neuroanat. 2015 Oct 9;9:129. doi: 10.3389/fnana.2015.00129 (PMC4598478; doi:10.3389/fnana.2015.00129)
Supplement: Supplementary file 1 [file Table_1.DOC]

Table S1. Detailed information of the loci used in this study

| SNP | Chr | Position | Gene | Subsystem | Maj | N | Het | N | Min | N | Miss | HWE p | LD  r^2^>0.8 | Deleted |
| --- | --- | --- | --- | --- | --- | --- | --- | --- | --- | --- | --- | --- | --- | --- |
| rs1800532 | 11 | 18004392 | TPH1 | Synthesis | CC | 123 | AC | 195 | AA | 99 | 0 | 0.45 |  |  |
| rs10488683 | 11 | 18010121 | TPH1 |  | AA | 153 | AG | 174 | GG | 90 | 0 | 0.01 |  |  |
| rs11024449 | 11 | 18014461 | TPH1 |  | GG | 396 | AG | 21 | AA |  | 0 | 0.59 |  |  |
| rs11179000 | 12 | 70624895 | TPH2 |  | AA | 304 | AT | 113 | TT |  | 0 | 0.00 |  |  |
| rs7955501 | 12 | 70636293 | TPH2 |  | AA | 154 | AT | 194 | TT | 69 | 0 | 0.64 |  |  |
| rs1487275 | 12 | 70696559 | TPH2 |  | AA | 174 | AC | 188 | CC | 55 | 0 | 0.93 |  |  |
| rs929095 | 23 | 43247894 | MAOA | Degradation | GG | 164 | CG | 253 | CC |  | 0 | 0.48 |  |  |
| rs1181286 | 23 | 43300472 | MAOA |  | CC | 347 | AC | 70 | AA |  | 0 | 0.52 | 1 |  |
| rs1181289 | 23 | 43325208 | MAOA |  | TT | 347 | AT | 70 | AA |  | 0 | 0.95 | 1 | Y |
| rs5906974 | 23 | 43436265 | MAOA |  | GG | 190 | AG | 227 | AA |  | 0 | 0.13 | 2 | Y |
| rs909525 | 23 | 43438146 | MAOA |  | GG | 190 | AG | 226 | AA |  | 1 | 0.13 | 2 |  |
| MAOA_VNTR | 23 |  | MAOA |  | 3/3 | 184 | 3/4 | 227 | 4/4 |  | 9 | 0.61 |  |  |
| rs1799836 | 23 | 43512943 | MAOB |  | AA | 306 | AG | 111 | GG |  | 0 | 0.22 |  |  |
| rs6651806 | 23 | 43573908 | MAOB |  | AA | 331 | AC | 86 | CC |  | 0 | 0.71 |  |  |
| rs5905512 | 23 | 43611338 | MAOB |  | AA | 247 | AG | 170 | GG |  | 0 | 0.17 |  |  |
| rs1042173 | 17 | 25549137 | SLC6A4 | Transport | CC | 266 | AC | 128 | AA | 23 | 0 | 0.13 | 3 | Y |
| rs4325622 | 17 | 25550601 | SLC6A4 |  | GG | 266 | AG | 128 | AA | 23 | 0 | 0.13 | 3 | Y |
| rs3794808 | 17 | 25555919 | SLC6A4 |  | AA | 269 | AG | 124 | GG | 24 | 0 | 0.05 | 3 | Y |
| rs140701 | 17 | 25562658 | SLC6A4 |  | AA | 267 | AG | 125 | GG | 25 | 0 | 0.04 | 3 |  |
| rs4583306 | 17 | 25562841 | SLC6A4 |  | GG | 268 | AG | 125 | AA | 24 | 0 | 0.05 | 3 | Y |
| rs2020942 | 17 | 25571040 | SLC6A4 |  | GG | 351 | AG | 66 | AA |  | 0 | 0.87 |  |  |
| rs8076005 | 17 | 25571336 | SLC6A4 |  | AA | 307 | AG | 100 | GG | 10 | 0 | 0.35 |  |  |
| 5HTTLPR | 17 |  | SLC6A4 |  | SS | 216 | SL | 161 | LL | 38 | 3 | 0.45 |  |  |
| rs1497020 | 8 | 20046706 | SLC18A1(VMAT1) |  | AA | 118 | AG | 200 | GG | 99 | 0 | 0.75 |  |  |
| rs1018079 | 8 | 20049580 | SLC18A1(VMAT1) |  | AA | 266 | AG | 135 | GG | 15 | 1 | 0.71 | 4 |  |
| rs12545707 | 8 | 20049620 | SLC18A1(VMAT1) |  | GG | 400 | AG | 17 | AA |  | 0 | 0.64 |  |  |
| rs903997 | 8 | 20049996 | SLC18A1(VMAT1) |  | GG | 124 | CG | 201 | CC | 92 | 0 | 0.84 |  |  |
| rs2270650 | 8 | 20052292 | SLC18A1(VMAT1) |  | GG | 280 | AG | 121 | AA | 16 | 0 | 0.46 | 4 | Y |
| rs3779671 | 8 | 20067165 | SLC18A1(VMAT1) |  | GG | 196 | CG | 160 | CC | 61 | 0 | 0.01 |  |  |
| rs13258461 | 8 | 20073883 | SLC18A1(VMAT1) |  | GG | 146 | AG | 191 | AA | 80 | 0 | 0.09 |  |  |
| rs952858 | 8 | 20076882 | SLC18A1(VMAT1) |  | GG | 379 | CG | 38 | CC |  | 0 | 0.00 |  |  |
| rs2270638 | 8 | 20081297 | SLC18A1(VMAT1) |  | AA | 300 | AG | 116 | GG |  | 1 | 0.07 |  |  |
| rs363338 | 10 | 118999379 | SLC18A2(VMAT2) |  | GG | 266 | AG | 140 | AA | 11 | 0 | 0.32 |  |  |
| rs363222 | 10 | 119009438 | SLC18A2(VMAT2) |  | CC | 180 | CG | 186 | GG | 49 | 2 | 0.85 |  |  |
| rs4752045 | 10 | 119009680 | SLC18A2(VMAT2) |  | GG | 132 | CG | 239 | CC | 46 | 0 | 0.00 |  |  |
| rs363225 | 10 | 119014492 | SLC18A2(VMAT2) |  | GG | 128 | AG | 208 | AA | 80 | 1 | 0.58 |  |  |
| rs363226 | 10 | 119015202 | SLC18A2(VMAT2) |  | CC | 349 | CG | 68 | GG |  | 0 | 0.18 |  |  |
| rs878567 | 5 | 63291747 | HTR1A | Receptor | GG | 270 | AG | 138 | AA | 9 | 0 | 0.09 | 5 |  |
| rs10042486 | 5 | 63297085 | HTR1A |  | AA | 270 | AG | 138 | GG | 9 | 0 | 0.09 | 5 | Y |
| rs6297 | 6 | 78228660 | HTR1B |  | AA | 341 | AG | 76 | GG |  | 0 | 0.59 |  |  |
| rs6298 | 6 | 78229711 | HTR1B |  | AA | 116 | AG | 197 | GG | 104 | 0 | 0.37 |  |  |
| rs2776822 | 1 | 23365369 | HTR1D |  | GG | 140 | AG | 209 | AA | 68 | 0 | 0.36 | 6 | Y |
| rs2746553 | 1 | 23365833 | HTR1D |  | AA | 140 | AG | 209 | GG | 68 | 0 | 0.36 | 6 | Y |
| rs2776815 | 1 | 23383171 | HTR1D |  | AA | 234 | AG | 157 | GG | 26 | 0 | 0.74 | 7 | Y |
| rs6704440 | 1 | 23385634 | HTR1D |  | CC | 292 | AC | 113 | AA | 12 | 0 | 0.97 |  |  |
| rs641032 | 1 | 23386567 | HTR1D |  | AA | 234 | AG | 157 | GG | 26 | 0 | 0.74 | 7 | Y |
| rs604030 | 1 | 23390814 | HTR1D |  | AA | 234 | AG | 157 | GG | 26 | 0 | 0.74 | 7 | Y |
| rs676643 | 1 | 23393927 | HTR1D |  | GG | 239 | AG | 152 | AA | 26 | 0 | 0.52 | 7 | Y |
| rs674386 | 1 | 23394422 | HTR1D |  | GG | 235 | AG | 156 | AA | 26 | 0 | 0.69 | 7 |  |
| rs2746561 | 1 | 23399139 | HTR1D |  | AA | 239 | AG | 152 | GG | 26 | 0 | 0.52 | 7 | Y |
| rs598438 | 1 | 23404150 | HTR1D |  | GG | 147 | AG | 208 | AA | 62 | 0 | 0.29 | 6 |  |
| rs1738475 | 1 | 23409478 | HTR1D |  | GG | 141 | CG | 209 | CC | 67 | 0 | 0.34 | 6 | Y |
| rs627304 | 1 | 23410142 | HTR1D |  | AA | 141 | AG | 209 | GG | 67 | 0 | 0.34 | 6 | Y |
| rs2903545 | 1 | 23413695 | HTR1D |  | AA | 141 | AG | 209 | GG | 67 | 0 | 0.34 | 6 | Y |
| rs7630716 | 3 | 88116161 | HTR1F |  | CC | 213 | CG | 170 | GG | 34 | 0 | 0.82 | 8 | Y |
| rs9863076 | 3 | 88116629 | HTR1F |  | CC | 213 | AC | 170 | AA | 34 | 0 | 0.82 | 8 | Y |
| rs1503433 | 3 | 88119890 | HTR1F |  | AA | 213 | AC | 170 | CC | 34 | 0 | 0.82 | 8 | Y |
| rs2016224 | 3 | 88124340 | HTR1F |  | AA | 213 | AG | 170 | GG | 34 | 0 | 0.82 | 8 | Y |
| rs7652406 | 3 | 88135928 | HTR1F |  | GG | 214 | AG | 169 | AA | 34 | 0 | 0.77 | 8 |  |
| rs7997012 | 13 | 46309986 | HTR2A |  | GG | 230 | AG | 150 | AA | 37 | 0 | 0.13 |  |  |
| rs977003 | 13 | 46313002 | HTR2A |  | AA | 263 | AC | 130 | CC | 24 | 0 | 0.13 |  |  |
| rs6561332 | 13 | 46317821 | HTR2A |  | CC | 361 | AC | 56 | AA |  | 0 | 0.73 |  |  |
| rs6561333 | 13 | 46318313 | HTR2A |  | GG | 131 | AG | 199 | AA | 87 | 0 | 0.57 |  |  |
| rs9567739 | 13 | 46322945 | HTR2A |  | CC | 128 | CG | 184 | GG | 105 | 0 | 0.06 | 9 | Y |
| rs655888 | 13 | 46326182 | HTR2A |  | GG | 134 | AG | 180 | AA | 102 | 1 | 0.03 | 9 |  |
| rs7984966 | 13 | 46327447 | HTR2A |  | AA | 381 | AG | 36 | GG |  | 0 | 0.30 |  |  |
| rs1885884 | 13 | 46328277 | HTR2A |  | GG | 230 | CG | 156 | CC | 31 | 0 | 0.46 |  |  |
| rs9526240 | 13 | 46329472 | HTR2A |  | GG | 401 | AG | 16 | AA |  | 0 | 0.62 | 10 |  |
| rs2224721 | 13 | 46330155 | HTR2A |  | CC | 186 | AC | 181 | AA | 50 | 0 | 0.82 |  |  |
| rs6561335 | 13 | 46331498 | HTR2A |  | AA | 403 | AG | 14 | GG |  | 0 | 0.66 | 10 | Y |
| rs1928042 | 13 | 46335217 | HTR2A |  | AA | 375 | AC | 42 | CC |  | 0 | 0.56 |  |  |
| rs2770293 | 13 | 46336975 | HTR2A |  | GG | 256 | AG | 139 | AA | 22 | 0 | 0.83 | 11 | Y |
| rs9534501 | 13 | 46337977 | HTR2A |  | GG | 263 | AG | 133 | AA | 21 | 0 | 0.63 | 11 |  |
| rs2770296 | 13 | 46338561 | HTR2A |  | AA | 230 | AG | 158 | GG | 29 | 0 | 0.88 |  |  |
| rs985933 | 13 | 46353864 | HTR2A |  | GG | 175 | AG | 183 | AA | 58 | 1 | 0.31 | 12 | Y |
| rs927544 | 13 | 46354052 | HTR2A |  | AA | 204 | AG | 169 | GG | 44 | 0 | 0.24 | 12 |  |
| rs1328684 | 13 | 46364231 | HTR2A |  | AA | 355 | AG | 62 | GG |  | 0 | 0.54 |  |  |
| rs2296973 | 13 | 46364782 | HTR2A |  | CC | 183 | AC | 182 | AA | 52 | 0 | 0.53 | 13 |  |
| rs2070040 | 13 | 46365627 | HTR2A |  | GG | 176 | AG | 185 | AA | 55 | 1 | 0.48 | 13 | Y |
| rs953451 | 15 | 36027756 | HTR2A |  | CC | 212 | CG | 177 | GG | 28 | 0 | 0.12 |  |  |
| rs17619600 | 2 | 231684704 | HTR2B |  | AA | 264 | AG | 142 | GG | 11 | 0 | 0.12 |  |  |
| rs6437000 | 2 | 231685771 | HTR2B |  | AA | 142 | AC | 207 | CC | 68 | 0 | 0.46 | 14 |  |
| rs10194776 | 2 | 231688263 | HTR2B |  | AA | 148 | AG | 204 | GG | 65 | 0 | 0.62 | 14 | Y |
| rs16827801 | 2 | 231689021 | HTR2B |  | AA | 108 | AG | 211 | GG | 98 | 0 | 0.71 |  |  |
| rs1549339 | 2 | 231691070 | HTR2B |  | AA | 145 | AG | 205 | GG | 67 | 0 | 0.58 | 14 | Y |
| rs17586428 | 2 | 231697099 | HTR2B |  | AA | 250 | AG | 155 | GG | 12 | 0 | 0.14 |  |  |
| rs2192371 | 23 | 113796095 | HTR2C |  | AA | 212 | AG | 113 | GG | 92 | 0 | 0.94 |  |  |
| rs6644065 | 23 | 113808442 | HTR2C |  | AA | 326 | AG | 58 | GG | 33 | 0 | 0.50 |  |  |
| rs4911871 | 23 | 113903396 | HTR2C |  | AA | 308 | AG | 63 | GG | 45 | 1 | 0.93 |  |  |
| rs2276302 | 11 | 113355350 | HTR3A |  | AA | 330 | AG | 86 | GG |  | 1 | 0.92 |  |  |
| rs11214769 | 11 | 113295878 | HTR3B |  | AA | 294 | AG | 106 | GG | 17 | 0 | 0.09 |  |  |
| rs1176746 | 11 | 113307811 | HTR3B |  | GG | 176 | AG | 196 | AA | 44 | 1 | 0.17 |  |  |
| rs6808122 | 3 | 185255515 | HTR3C |  | AA | 236 | AG | 161 | GG | 20 | 0 | 0.54 |  |  |
| rs6766410 | 3 | 185257456 | HTR3C |  | AA | 158 | AC | 206 | CC | 53 | 0 | 0.14 |  |  |
| rs6807362 | 3 | 185260704 | HTR3C |  | GG | 211 | CG | 177 | CC | 29 | 0 | 0.25 |  |  |
| rs939334 | 3 | 185232425 | HTR3D |  | AA | 216 | AG | 161 | GG | 39 | 1 | 0.47 |  |  |
| rs10937159 | 3 | 185234995 | HTR3D |  | AA | 212 | AC | 158 | CC | 47 | 0 | 0.03 |  |  |
| rs6792482 | 3 | 185236723 | HTR3D |  | GG | 106 | AG | 209 | AA | 102 | 0 | 0.79 |  |  |
| rs1467257 | 3 | 185238225 | HTR3D |  | CC | 255 | AC | 129 | AA | 24 | 12 | 0.18 |  |  |
| rs7627615 | 3 | 185301110 | HTR3E |  | AA | 246 | AG | 150 | GG | 21 | 0 | 0.62 |  |  |
| rs7432211 | 3 | 185301849 | HTR3E |  | AA | 165 | AG | 204 | GG | 48 | 0 | 0.17 |  |  |
| rs7733401 | 5 | 147813474 | HTR4 |  | CC | 111 | AC | 226 | AA | 80 | 0 | 0.02 |  |  |
| rs3995090 | 5 | 147826008 | HTR4 |  | CC | 232 | AC | 156 | AA | 29 | 0 | 0.41 |  |  |
| rs4597955 | 5 | 147827466 | HTR4 |  | AA | 375 | AG | 42 | GG |  | 0 | 0.45 |  |  |
| rs10051356 | 5 | 147845252 | HTR4 |  | GG | 151 | CG | 207 | CC | 59 | 0 | 0.65 |  |  |
| rs1883074 | 5 | 147853943 | HTR4 |  | GG | 129 | AG | 212 | AA | 76 | 0 | 0.38 |  |  |
| rs13166761 | 5 | 147927481 | HTR4 |  | GG | 212 | AG | 175 | AA | 30 | 0 | 0.25 |  |  |
| rs6873382 | 5 | 147938456 | HTR4 |  | AA | 132 | AG | 214 | GG | 71 | 0 | 0.21 |  |  |
| rs7711800 | 5 | 147963048 | HTR4 |  | GG | 108 | AG | 215 | AA | 94 | 0 | 0.83 |  |  |
| rs6580561 | 5 | 147990262 | HTR4 |  | AA | 204 | AG | 174 | GG | 39 | 0 | 0.56 |  |  |
| rs1972644 | 5 | 147999741 | HTR4 |  | AA | 147 | AG | 191 | GG | 76 | 3 | 0.28 |  |  |
| rs1800883 | 7 | 154493524 | HTR5A |  | CC | 125 | CG | 188 | GG | 103 | 1 | 0.08 |  |  |
| rs6320 | 7 | 154493554 | HTR5A |  | AA | 172 | AT | 190 | TT | 55 | 0 | 0.95 |  |  |
| rs732050 | 7 | 154504328 | HTR5A |  | AA | 264 | AG | 135 | GG | 18 | 0 | 0.80 |  |  |
| rs1440449 | 7 | 154506190 | HTR5A |  | AA | 166 | AC | 188 | CC | 63 | 0 | 0.68 |  |  |
| rs11676829 | 2 | 118371773 | HTR5B |  | AA | 310 | AG | 99 | GG | 8 | 0 | 0.53 |  |  |
| rs2245686 | 2 | 118371917 | HTR5B |  | GG | 243 | AG | 143 | AA | 30 | 1 | 0.11 |  |  |
| rs10917509 | 1 | 19864653 | HTR6 |  | AA | 269 | AG | 148 | GG |  | 0 | 0.00 | 15 |  |
| rs4912138 | 1 | 19866072 | HTR6 |  | AA | 121 | AG | 187 | GG | 109 | 0 | 0.04 |  |  |
| rs6658108 | 1 | 19868078 | HTR6 |  | GG | 247 | AG | 143 | AA | 27 | 0 | 0.48 |  |  |
| rs3790757 | 1 | 19870495 | HTR6 |  | GG | 307 | AG | 110 | AA |  | 0 | 0.68 |  |  |
| rs9659997 | 1 | 19875105 | HTR6 |  | GG | 263 | AG | 135 | AA | 19 | 0 | 0.80 | 15 | Y |
| rs7904569 | 10 | 15650570 | HTR7 |  | AA | 182 | AG | 185 | GG | 50 | 0 | 0.94 |  |  |
| rs4933194 | 10 | 92501347 | HTR7 |  | GG | 173 | AG | 193 | AA | 51 | 0 | 0.52 |  |  |
| rs11596518 | 10 | 92544991 | HTR7 |  | AA | 264 | AG | 136 | GG | 17 | 0 | 0.93 |  |  |
| rs1573935 | 10 | 92554807 | HTR7 |  | AA | 115 | AG | 209 | GG | 93 | 0 | 0.80 |  |  |
| rs10881838 | 10 | 92580213 | HTR7 |  | AA | 143 | AG | 197 | GG | 77 | 0 | 0.83 |  |  |
| rs12249377 | 10 | 92582936 | HTR7 |  | CC | 298 | AC | 109 | AA | 10 | 0 | 0.76 |  |  |
| rs10785973 | 10 | 92588151 | HTR7 |  | CC | 213 | AC | 170 | AA | 34 | 0 | 0.57 |  |  |

Note: a. Chr = Chromosome, Maj= major homozygote, N= number of subjects, Het=heterozygote, Min=minor homozygote, Miss = number of subjects missing genotype.

b. SNPs with the same numbers in “LD” column are clusters with high LD (r^2^>0.8), “Y” in “deleted” column means this SNP was deleted because of high LD.

Table S2. Means and standard deviations of amygdala volume for each polymorphism, and main effects and post hoc comparisons of each locus.

| SNP | Subsystem | Gene | Maj | Mean | SD | n | Het | Mean | SD | n | Min | Mean | SD | n | F | p | mh | mm | hm |
| --- | --- | --- | --- | --- | --- | --- | --- | --- | --- | --- | --- | --- | --- | --- | --- | --- | --- | --- | --- |
| rs1800532 | Synthesis | TPH1 | CC | 1737.22 | 190.46 | 123 | AC | 1716.04 | 179.87 | 195 | AA | 1724.40 | 205.74 | 99 | 0.22 | 0.80 | 0.86 | 0.52 | 0.60 |
| rs10488683 |  | TPH1 | AA | 1718.43 | 190.68 | 153 | AG | 1727.48 | 190.27 | 174 | GG | 1727.99 | 186.21 | 90 | 0.67 | 0.51 | 0.29 | 0.94 | 0.40 |
| rs11024449 |  | TPH1 | GG | 1722.78 | 190.24 | 396 | AG | 1752.40 | 169.06 | 21 | AA |  |  |  | 0.04 | 0.84 | 0.84 |  |  |
| rs11179000 |  | TPH2 | AA | 1720.35 | 181.93 | 304 | AT | 1734.82 | 207.84 | 113 | TT |  |  |  | 1.17 | 0.28 | 0.28 |  |  |
| rs7955501 |  | TPH2 | AA | 1717.71 | 186.14 | 154 | AT | 1721.46 | 194.07 | 194 | TT | 1746.80 | 182.72 | 69 | 1.29 | 0.28 | 0.73 | 0.12 | 0.18 |
| rs1487275 |  | TPH2 | AA | 1723.66 | 187.81 | 174 | AC | 1709.73 | 181.82 | 188 | CC | 1775.90 | 211.43 | 55 | 3.68 | 0.03 | 0.38 | 0.04 | 0.01 |
| rs929095 | Degradation | MAOA | GG | 1740.34 | 179.02 | 164 | CG | 1713.85 | 195.08 | 253 | CC |  |  |  | 0.12 | 0.73 | 0.73 |  |  |
| rs1181286 |  | MAOA | CC | 1725.54 | 191.42 | 347 | AC | 1717.97 | 178.67 | 70 | AA |  |  |  | 0.07 | 0.79 | 0.79 |  |  |
| rs1181289 |  | MAOA | TT | 1725.54 | 191.42 | 347 | AT | 1717.97 | 178.67 | 70 | AA |  |  |  | 0.07 | 0.79 | 0.79 |  |  |
| rs5906974 |  | MAOA | GG | 1733.55 | 197.92 | 190 | AG | 1716.51 | 181.56 | 227 | AA |  |  |  | 0.00 | 0.99 | 0.99 |  |  |
| rs909525 |  | MAOA | GG | 1733.55 | 197.92 | 190 | AG | 1717.32 | 181.55 | 226 | AA |  |  |  | 0.00 | 0.96 | 0.96 |  |  |
| MAOA_VNTR |  | MAOA | 3 | 1737.91 | 188.36 | 184 |  | 1712.31 | 188.69 | 227 | 4 |  |  |  | 0.08 | 0.78 | 0.78 |  |  |
| rs1799836 |  | MAOB | AA | 1723.44 | 196.88 | 306 | AG | 1726.57 | 166.83 | 111 | GG |  |  |  | 1.02 | 0.31 | 0.31 |  |  |
| rs6651806 |  | MAOB | AA | 1735.12 | 190.31 | 331 | AC | 1682.51 | 179.64 | 86 | CC |  |  |  | 0.77 | 0.38 | 0.38 |  |  |
| rs5905512 |  | MAOB | AA | 1730.35 | 199.08 | 247 | AG | 1715.43 | 173.92 | 170 | GG |  |  |  | 0.61 | 0.43 | 0.43 |  |  |
| rs1042173 | Transport | SLC6A4 | CC | 1717.05 | 185.62 | 266 | AC | 1740.58 | 189.60 | 128 | AA | 1717.07 | 227.52 | 23 | 0.14 | 0.87 | 0.91 | 0.63 | 0.61 |
| rs4325622 |  | SLC6A4 | GG | 1717.05 | 185.62 | 266 | AG | 1740.58 | 189.60 | 128 | AA | 1717.07 | 227.52 | 23 | 0.14 | 0.87 | 0.91 | 0.63 | 0.61 |
| rs3794808 |  | SLC6A4 | AA | 1717.57 | 185.18 | 269 | AG | 1741.22 | 189.03 | 124 | GG | 1711.85 | 232.86 | 24 | 0.11 | 0.90 | 0.95 | 0.66 | 0.65 |
| rs140701 |  | SLC6A4 | AA | 1715.81 | 186.37 | 267 | AG | 1747.69 | 183.39 | 125 | GG | 1697.61 | 238.82 | 25 | 0.58 | 0.56 | 0.53 | 0.45 | 0.30 |
| rs4583306 |  | SLC6A4 | GG | 1715.04 | 186.45 | 268 | AG | 1748.85 | 182.33 | 125 | AA | 1699.37 | 243.79 | 24 | 0.50 | 0.61 | 0.50 | 0.54 | 0.36 |
| rs2020942 |  | SLC6A4 | GG | 1729.04 | 190.33 | 351 | AG | 1698.88 | 182.06 | 66 | AA |  |  |  | 1.37 | 0.24 | 0.24 |  |  |
| rs8076005 |  | SLC6A4 | AA | 1714.76 | 184.19 | 307 | AG | 1747.55 | 198.08 | 100 | GG | 1783.59 | 235.85 | 10 | 0.51 | 0.60 | 0.54 | 0.39 | 0.54 |
| 5HTTLPR |  | SLC6A4 | SS | 1732.70 | 179.42 | 216 | LS | 1709.34 | 195.25 | 161 | LL | 1725.93 | 205.33 | 38 | 1.36 | 0.26 | 0.10 | 0.79 | 0.49 |
| rs1497020 |  | SLC18A1(VMAT1) | AA | 1730.51 | 187.41 | 118 | AG | 1707.15 | 189.29 | 200 | GG | 1751.43 | 189.21 | 99 | 0.76 | 0.47 | 0.88 | 0.34 | 0.23 |
| rs1018079 |  | SLC18A1(VMAT1) | AA | 1739.26 | 188.74 | 266 | AG | 1696.93 | 192.95 | 135 | GG | 1703.92 | 143.40 | 15 | 1.50 | 0.22 | 0.09 | 0.61 | 0.87 |
| rs12545707 |  | SLC18A1(VMAT1) | GG | 1723.73 | 187.96 | 400 | AG | 1736.96 | 221.49 | 17 | AA |  |  |  | 1.64 | 0.20 | 0.20 |  |  |
| rs903997 |  | SLC18A1(VMAT1) | GG | 1721.42 | 183.52 | 124 | CG | 1714.05 | 193.10 | 201 | CC | 1750.44 | 187.49 | 92 | 0.74 | 0.48 | 0.65 | 0.23 | 0.36 |
| rs2270650 |  | SLC18A1(VMAT1) | GG | 1734.39 | 188.28 | 280 | AG | 1704.21 | 196.05 | 121 | AA | 1698.81 | 140.04 | 16 | 0.46 | 0.63 | 0.37 | 0.65 | 0.94 |
| rs3779671 |  | SLC18A1(VMAT1) | GG | 1723.72 | 189.68 | 196 | CG | 1715.82 | 194.33 | 160 | CC | 1748.20 | 174.01 | 61 | 1.02 | 0.36 | 0.59 | 0.28 | 0.15 |
| rs13258461 |  | SLC18A1(VMAT1) | GG | 1716.11 | 188.72 | 146 | AG | 1717.09 | 191.46 | 191 | AA | 1756.30 | 183.28 | 80 | 2.25 | 0.11 | 0.98 | 0.06 | 0.05 |
| rs952858 |  | SLC18A1(VMAT1) | GG | 1721.39 | 191.65 | 379 | CG | 1752.95 | 161.56 | 38 | CC |  |  |  | 1.74 | 0.19 | 0.19 |  |  |
| rs2270638 |  | SLC18A1(VMAT1) | AA | 1725.32 | 186.12 | 300 | AG | 1722.45 | 198.15 | 116 | GG |  |  |  | 0.06 | 0.81 | 0.81 |  |  |
| rs363338 |  | SLC18A2(VMAT2) | GG | 1730.63 | 194.11 | 266 | AG | 1719.13 | 180.15 | 140 | AA | 1635.81 | 169.63 | 11 | 0.07 | 0.93 | 1.00 | 0.71 | 0.72 |
| rs363222 |  | SLC18A2(VMAT2) | CC | 1739.65 | 180.28 | 180 | CG | 1710.55 | 194.53 | 186 | GG | 1724.22 | 201.67 | 49 | 1.97 | 0.14 | 0.05 | 0.66 | 0.40 |
| rs4752045 |  | SLC18A2(VMAT2) | GG | 1721.68 | 193.20 | 132 | CG | 1730.53 | 187.63 | 239 | CC | 1699.19 | 187.17 | 46 | 0.22 | 0.80 | 0.55 | 1.00 | 0.68 |
| rs363225 |  | SLC18A2(VMAT2) | GG | 1736.63 | 200.28 | 128 | AG | 1709.44 | 185.52 | 208 | AA | 1740.13 | 178.77 | 80 | 2.55 | 0.08 | 0.05 | 0.95 | 0.09 |
| rs363226 |  | SLC18A2(VMAT2) | CC | 1735.34 | 186.30 | 349 | CG | 1667.48 | 194.85 | 68 | GG |  |  |  | 8.99 | 0.00 | 0.00 |  |  |
| rs878567 | Receptor | HTR1A | GG | 1717.57 | 188.41 | 270 | AG | 1735.30 | 186.54 | 138 | AA | 1756.09 | 257.36 | 9 | 0.25 | 0.78 | 0.61 | 0.58 | 0.70 |
| rs10042486 |  | HTR1A | AA | 1717.57 | 188.41 | 270 | AG | 1735.30 | 186.54 | 138 | GG | 1756.09 | 257.36 | 9 | 0.25 | 0.78 | 0.61 | 0.58 | 0.70 |
| rs6297 |  | HTR1B | AA | 1727.09 | 190.17 | 341 | AG | 1711.62 | 185.24 | 76 | GG |  |  |  | 0.39 | 0.53 | 0.53 |  |  |
| rs6298 |  | HTR1B | AA | 1739.90 | 195.30 | 116 | AG | 1718.43 | 193.33 | 197 | GG | 1717.91 | 174.39 | 104 | 0.02 | 0.98 | 0.85 | 1.00 | 0.86 |
| rs2776822 |  | HTR1D | GG | 1734.57 | 190.20 | 140 | AG | 1715.92 | 192.58 | 209 | AA | 1728.74 | 177.49 | 68 | 1.59 | 0.21 | 0.39 | 0.31 | 0.08 |
| rs2746553 |  | HTR1D | AA | 1734.57 | 190.20 | 140 | AG | 1715.92 | 192.58 | 209 | GG | 1728.74 | 177.49 | 68 | 1.59 | 0.21 | 0.39 | 0.31 | 0.08 |
| rs2776815 |  | HTR1D | AA | 1733.20 | 196.54 | 234 | AG | 1713.32 | 179.23 | 157 | GG | 1710.06 | 182.18 | 26 | 0.41 | 0.66 | 0.49 | 0.66 | 0.44 |
| rs6704440 |  | HTR1D | CC | 1720.05 | 185.09 | 292 | AC | 1732.83 | 201.06 | 113 | AA | 1746.32 | 182.90 | 12 | 0.56 | 0.57 | 0.51 | 0.37 | 0.53 |
| rs641032 |  | HTR1D | AA | 1733.20 | 196.54 | 234 | AG | 1713.32 | 179.23 | 157 | GG | 1710.06 | 182.18 | 26 | 0.41 | 0.66 | 0.49 | 0.66 | 0.44 |
| rs604030 |  | HTR1D | AA | 1733.20 | 196.54 | 234 | AG | 1713.32 | 179.23 | 157 | GG | 1710.06 | 182.18 | 26 | 0.41 | 0.66 | 0.49 | 0.66 | 0.44 |
| rs676643 |  | HTR1D | GG | 1732.87 | 194.71 | 239 | AG | 1713.17 | 181.78 | 152 | AA | 1710.06 | 182.18 | 26 | 0.36 | 0.70 | 0.55 | 0.65 | 0.46 |
| rs674386 |  | HTR1D | GG | 1733.15 | 196.12 | 235 | AG | 1713.26 | 179.80 | 156 | AA | 1710.06 | 182.18 | 26 | 0.42 | 0.66 | 0.49 | 0.66 | 0.44 |
| rs2746561 |  | HTR1D | AA | 1732.87 | 194.71 | 239 | AG | 1713.17 | 181.78 | 152 | GG | 1710.06 | 182.18 | 26 | 0.36 | 0.70 | 0.55 | 0.65 | 0.46 |
| rs598438 |  | HTR1D | GG | 1739.47 | 191.66 | 147 | AG | 1713.61 | 189.83 | 208 | AA | 1723.99 | 181.24 | 62 | 1.59 | 0.21 | 0.21 | 0.53 | 0.11 |
| rs1738475 |  | HTR1D | GG | 1737.33 | 191.27 | 141 | CG | 1715.04 | 191.59 | 209 | CC | 1725.60 | 177.74 | 67 | 1.49 | 0.23 | 0.28 | 0.46 | 0.11 |
| rs627304 |  | HTR1D | AA | 1737.33 | 191.27 | 141 | AG | 1715.04 | 191.59 | 209 | GG | 1725.60 | 177.74 | 67 | 1.49 | 0.23 | 0.28 | 0.46 | 0.11 |
| rs2903545 |  | HTR1D | AA | 1737.33 | 191.27 | 141 | AG | 1715.04 | 191.59 | 209 | GG | 1725.60 | 177.74 | 67 | 1.49 | 0.23 | 0.28 | 0.46 | 0.11 |
| rs7630716 |  | HTR1F | CC | 1730.88 | 178.07 | 213 | CG | 1721.26 | 196.54 | 170 | GG | 1697.91 | 220.16 | 34 | 1.95 | 0.14 | 0.32 | 0.06 | 0.19 |
| rs9863076 |  | HTR1F | CC | 1730.88 | 178.07 | 213 | AC | 1721.26 | 196.54 | 170 | AA | 1697.91 | 220.16 | 34 | 1.95 | 0.14 | 0.32 | 0.06 | 0.19 |
| rs1503433 |  | HTR1F | AA | 1730.88 | 178.07 | 213 | AC | 1721.26 | 196.54 | 170 | CC | 1697.91 | 220.16 | 34 | 1.95 | 0.14 | 0.32 | 0.06 | 0.19 |
| rs2016224 |  | HTR1F | AA | 1730.88 | 178.07 | 213 | AG | 1721.26 | 196.54 | 170 | GG | 1697.91 | 220.16 | 34 | 1.95 | 0.14 | 0.32 | 0.06 | 0.19 |
| rs7652406 |  | HTR1F | GG | 1730.51 | 177.73 | 214 | AG | 1721.68 | 197.05 | 169 | AA | 1697.91 | 220.16 | 34 | 1.96 | 0.14 | 0.31 | 0.06 | 0.19 |
| rs7997012 |  | HTR2A | GG | 1718.46 | 196.29 | 230 | AG | 1713.51 | 170.93 | 150 | AA | 1804.02 | 200.47 | 37 | 3.87 | 0.02 | 0.39 | 0.02 | 0.01 |
| rs977003 |  | HTR2A | AA | 1722.37 | 196.15 | 263 | AC | 1713.97 | 175.63 | 130 | CC | 1800.93 | 170.54 | 24 | 1.08 | 0.34 | 0.26 | 0.45 | 0.21 |
| rs6561332 |  | HTR2A | CC | 1723.03 | 190.68 | 361 | AC | 1732.28 | 180.41 | 56 | AA |  |  |  | 0.21 | 0.65 | 0.65 |  |  |
| rs6561333 |  | HTR2A | GG | 1718.94 | 184.01 | 131 | AG | 1714.54 | 189.54 | 199 | AA | 1754.56 | 194.90 | 87 | 1.04 | 0.36 | 0.55 | 0.39 | 0.15 |
| rs9567739 |  | HTR2A | CC | 1740.40 | 193.08 | 128 | CG | 1714.65 | 195.65 | 184 | GG | 1721.48 | 172.46 | 105 | 0.19 | 0.83 | 0.68 | 0.85 | 0.56 |
| rs655888 |  | HTR2A | GG | 1741.34 | 196.43 | 134 | AG | 1711.09 | 192.42 | 180 | AA | 1723.48 | 173.11 | 102 | 0.38 | 0.68 | 0.47 | 0.96 | 0.47 |
| rs7984966 |  | HTR2A | AA | 1731.00 | 190.66 | 381 | AG | 1653.04 | 157.77 | 36 | GG |  |  |  | 6.48 | 0.01 | 0.01 |  |  |
| rs1885884 |  | HTR2A | GG | 1723.40 | 189.61 | 230 | CG | 1726.75 | 192.23 | 156 | CC | 1718.20 | 175.21 | 31 | 0.06 | 0.94 | 0.77 | 0.93 | 0.81 |
| rs9526240 |  | HTR2A | GG | 1722.82 | 190.49 | 401 | AG | 1760.74 | 152.05 | 16 | AA |  |  |  | 1.77 | 0.18 | 0.18 |  |  |
| rs2224721 |  | HTR2A | CC | 1715.45 | 190.36 | 186 | AC | 1729.09 | 192.74 | 181 | AA | 1739.63 | 172.79 | 50 | 0.58 | 0.56 | 0.48 | 0.32 | 0.59 |
| rs6561335 |  | HTR2A | AA | 1723.94 | 190.48 | 403 | AG | 1733.78 | 151.23 | 14 | GG |  |  |  | 0.12 | 0.73 | 0.73 |  |  |
| rs1928042 |  | HTR2A | AA | 1724.89 | 189.30 | 375 | AC | 1718.71 | 190.06 | 42 | CC |  |  |  | 0.08 | 0.78 | 0.78 |  |  |
| rs2770293 |  | HTR2A | GG | 1719.10 | 189.06 | 256 | AG | 1736.24 | 191.77 | 139 | AA | 1708.85 | 177.54 | 22 | 0.43 | 0.65 | 0.37 | 0.95 | 0.63 |
| rs9534501 |  | HTR2A | GG | 1716.80 | 187.59 | 263 | AG | 1743.21 | 194.25 | 133 | AA | 1697.86 | 174.08 | 21 | 0.91 | 0.40 | 0.21 | 0.75 | 0.38 |
| rs2770296 |  | HTR2A | AA | 1716.58 | 184.78 | 230 | AG | 1738.11 | 200.82 | 158 | GG | 1709.83 | 156.29 | 29 | 0.97 | 0.38 | 0.17 | 0.99 | 0.49 |
| rs985933 |  | HTR2A | GG | 1712.25 | 177.62 | 175 | AG | 1736.18 | 191.94 | 183 | AA | 1723.44 | 215.12 | 58 | 1.20 | 0.30 | 0.14 | 0.33 | 0.96 |
| rs927544 |  | HTR2A | AA | 1709.05 | 178.11 | 204 | AG | 1747.67 | 191.79 | 169 | GG | 1704.94 | 221.45 | 44 | 1.84 | 0.16 | 0.06 | 0.76 | 0.38 |
| rs1328684 |  | HTR2A | AA | 1727.26 | 193.09 | 355 | AG | 1707.16 | 165.18 | 62 | GG |  |  |  | 0.13 | 0.72 | 0.72 |  |  |
| rs2296973 |  | HTR2A | CC | 1708.23 | 175.55 | 183 | AC | 1741.60 | 191.99 | 182 | AA | 1720.09 | 221.44 | 52 | 1.37 | 0.26 | 0.10 | 0.56 | 0.60 |
| rs2070040 |  | HTR2A | GG | 1709.43 | 175.03 | 176 | AG | 1740.77 | 192.19 | 185 | AA | 1721.15 | 217.92 | 55 | 1.23 | 0.29 | 0.12 | 0.54 | 0.65 |
| rs953451 |  | HTR2A | CC | 1731.61 | 194.48 | 212 | CG | 1716.42 | 177.38 | 177 | GG | 1718.33 | 222.99 | 28 | 1.38 | 0.25 | 0.10 | 0.61 | 0.74 |
| rs17619600 |  | HTR2B | AA | 1725.62 | 185.57 | 264 | AG | 1726.89 | 199.35 | 142 | GG | 1658.02 | 132.02 | 11 | 1.84 | 0.16 | 0.58 | 0.08 | 0.06 |
| rs6437000 |  | HTR2B | AA | 1734.92 | 189.08 | 142 | AC | 1704.01 | 179.59 | 207 | CC | 1763.69 | 211.33 | 68 | 2.27 | 0.10 | 0.13 | 0.49 | 0.06 |
| rs10194776 |  | HTR2B | AA | 1732.11 | 187.51 | 148 | AG | 1704.30 | 179.78 | 204 | GG | 1769.10 | 214.07 | 65 | 2.09 | 0.12 | 0.26 | 0.28 | 0.05 |
| rs16827801 |  | HTR2B | AA | 1738.46 | 185.70 | 108 | AG | 1712.37 | 190.47 | 211 | GG | 1734.26 | 190.35 | 98 | 0.24 | 0.79 | 0.51 | 0.85 | 0.68 |
| rs1549339 |  | HTR2B | AA | 1731.01 | 189.26 | 145 | AG | 1709.75 | 185.82 | 205 | GG | 1754.10 | 197.43 | 67 | 1.12 | 0.33 | 0.27 | 0.65 | 0.19 |
| rs17586428 |  | HTR2B | AA | 1728.24 | 193.48 | 250 | AG | 1717.72 | 181.00 | 155 | GG | 1726.18 | 214.73 | 12 | 0.65 | 0.52 | 0.26 | 0.91 | 0.78 |
| rs2192371 |  | HTR2C | AA | 1738.03 | 185.36 | 212 | AG | 1665.14 | 174.08 | 113 | GG | 1765.20 | 200.31 | 92 | 0.03 | 0.97 | 0.81 | 0.90 | 0.93 |
| rs6644065 |  | HTR2C | AA | 1729.12 | 185.85 | 326 | AG | 1654.39 | 191.15 | 58 | GG | 1799.17 | 185.37 | 33 | 0.10 | 0.90 | 0.85 | 0.70 | 0.66 |
| rs4911871 |  | HTR2C | AA | 1726.55 | 188.04 | 308 | AG | 1653.72 | 184.09 | 63 | GG | 1810.19 | 169.51 | 45 | 0.68 | 0.51 | 0.63 | 0.33 | 0.25 |
| rs2276302 |  | HTR3A | AA | 1723.01 | 192.51 | 330 | AG | 1730.35 | 177.44 | 86 | GG |  |  |  | 0.08 | 0.77 | 0.77 |  |  |
| rs11214769 |  | HTR3B | AA | 1721.56 | 192.98 | 294 | AG | 1725.92 | 178.51 | 106 | GG | 1760.86 | 193.78 | 17 | 0.70 | 0.50 | 0.58 | 0.27 | 0.41 |
| rs1176746 |  | HTR3B | GG | 1727.94 | 188.51 | 176 | AG | 1712.46 | 192.83 | 196 | AA | 1761.47 | 175.41 | 44 | 0.62 | 0.54 | 0.49 | 0.55 | 0.30 |
| rs6808122 |  | HTR3C | AA | 1723.56 | 190.42 | 236 | AG | 1726.35 | 185.39 | 161 | GG | 1715.89 | 212.98 | 20 | 0.04 | 0.96 | 0.97 | 0.77 | 0.79 |
| rs6766410 |  | HTR3C | AA | 1738.10 | 213.20 | 158 | AC | 1710.28 | 170.73 | 206 | CC | 1737.40 | 180.42 | 53 | 1.69 | 0.19 | 0.07 | 0.58 | 0.49 |
| rs6807362 |  | HTR3C | GG | 1732.54 | 197.89 | 211 | CG | 1715.05 | 180.86 | 177 | CC | 1720.38 | 176.63 | 29 | 0.96 | 0.38 | 0.17 | 0.92 | 0.55 |
| rs939334 |  | HTR3D | AA | 1739.33 | 180.99 | 216 | AG | 1722.04 | 200.46 | 161 | GG | 1655.10 | 172.98 | 39 | 3.54 | 0.03 | 0.19 | 0.01 | 0.08 |
| rs10937159 |  | HTR3D | AA | 1722.42 | 191.12 | 212 | AC | 1737.36 | 192.91 | 158 | CC | 1688.61 | 164.63 | 47 | 0.76 | 0.47 | 0.43 | 0.49 | 0.24 |
| rs6792482 |  | HTR3D | GG | 1689.61 | 180.70 | 106 | AG | 1729.74 | 184.16 | 209 | AA | 1749.08 | 204.04 | 102 | 4.38 | 0.01 | 0.03 | 0.00 | 0.22 |
| rs1467257 |  | HTR3D | CC | 1726.93 | 196.69 | 255 | AC | 1726.95 | 185.06 | 129 | AA | 1707.88 | 146.78 | 24 | 0.07 | 0.93 | 0.73 | 0.93 | 0.80 |
| rs7627615 |  | HTR3E | AA | 1730.62 | 196.69 | 246 | AG | 1716.28 | 179.45 | 150 | GG | 1707.00 | 170.52 | 21 | 0.28 | 0.76 | 0.54 | 0.61 | 0.82 |
| rs7432211 |  | HTR3E | AA | 1737.01 | 203.69 | 165 | AG | 1718.31 | 180.62 | 204 | GG | 1705.81 | 173.00 | 48 | 0.63 | 0.53 | 0.60 | 0.27 | 0.43 |
| rs7733401 |  | HTR4 | CC | 1750.78 | 157.88 | 111 | AC | 1717.86 | 205.90 | 226 | AA | 1705.60 | 177.48 | 80 | 1.29 | 0.28 | 0.21 | 0.13 | 0.55 |
| rs3995090 |  | HTR4 | CC | 1709.99 | 194.77 | 232 | AC | 1729.87 | 178.42 | 156 | AA | 1808.36 | 182.24 | 29 | 1.72 | 0.18 | 0.61 | 0.07 | 0.12 |
| rs4597955 |  | HTR4 | AA | 1722.45 | 188.21 | 375 | AG | 1740.50 | 198.98 | 42 | GG |  |  |  | 0.01 | 0.92 | 0.92 |  |  |
| rs10051356 |  | HTR4 | GG | 1736.72 | 187.53 | 151 | CG | 1717.22 | 189.65 | 207 | CC | 1717.14 | 193.09 | 59 | 0.07 | 0.93 | 0.71 | 0.83 | 0.96 |
| rs1883074 |  | HTR4 | GG | 1715.50 | 183.51 | 129 | AG | 1719.40 | 199.23 | 212 | AA | 1752.75 | 168.21 | 76 | 1.05 | 0.35 | 0.37 | 0.15 | 0.42 |
| rs13166761 |  | HTR4 | GG | 1736.63 | 193.94 | 212 | AG | 1711.25 | 187.29 | 175 | AA | 1712.87 | 163.84 | 30 | 0.78 | 0.46 | 0.94 | 0.23 | 0.22 |
| rs6873382 |  | HTR4 | AA | 1712.42 | 190.07 | 132 | AG | 1729.01 | 195.65 | 214 | GG | 1732.00 | 167.76 | 71 | 0.08 | 0.92 | 0.74 | 0.73 | 0.91 |
| rs7711800 |  | HTR4 | GG | 1735.76 | 183.66 | 108 | AG | 1726.01 | 189.96 | 215 | AA | 1707.10 | 194.27 | 94 | 0.51 | 0.60 | 0.63 | 0.32 | 0.50 |
| rs6580561 |  | HTR4 | AA | 1722.08 | 189.75 | 204 | AG | 1718.33 | 181.15 | 174 | GG | 1762.25 | 219.74 | 39 | 1.50 | 0.22 | 0.67 | 0.13 | 0.08 |
| rs1972644 |  | HTR4 | AA | 1720.69 | 197.04 | 147 | AG | 1714.09 | 176.68 | 191 | GG | 1760.00 | 197.73 | 76 | 2.83 | 0.06 | 0.24 | 0.18 | 0.02 |
| rs1800883 |  | HTR5A | CC | 1728.62 | 192.60 | 125 | CG | 1717.84 | 183.46 | 188 | GG | 1731.76 | 197.12 | 103 | 0.65 | 0.52 | 0.52 | 0.64 | 0.27 |
| rs6320 |  | HTR5A | AA | 1733.34 | 195.16 | 172 | AT | 1712.81 | 176.28 | 190 | TT | 1735.49 | 213.10 | 55 | 0.37 | 0.69 | 0.39 | 0.77 | 0.77 |
| rs732050 |  | HTR5A | AA | 1718.63 | 191.91 | 264 | AG | 1733.31 | 184.88 | 135 | GG | 1739.23 | 186.94 | 18 | 0.16 | 0.85 | 0.60 | 0.78 | 0.96 |
| rs1440449 |  | HTR5A | AA | 1725.91 | 183.92 | 166 | AC | 1722.85 | 196.39 | 188 | CC | 1724.19 | 183.55 | 63 | 0.24 | 0.79 | 0.50 | 0.71 | 0.90 |
| rs11676829 |  | HTR5B | AA | 1715.14 | 186.37 | 310 | AG | 1737.48 | 186.99 | 99 | GG | 1914.46 | 236.80 | 8 | 3.71 | 0.03 | 0.11 | 0.02 | 0.08 |
| rs2245686 |  | HTR5B | GG | 1732.17 | 193.85 | 243 | AG | 1715.25 | 187.10 | 143 | AA | 1709.53 | 160.91 | 30 | 0.46 | 0.63 | 0.61 | 0.37 | 0.55 |
| rs10917509 |  | HTR6 | AA | 1738.57 | 190.66 | 269 | AG | 1698.28 | 184.18 | 148 | GG |  |  |  | 5.38 | 0.02 | 0.02 |  |  |
| rs4912138 |  | HTR6 | AA | 1737.20 | 184.15 | 121 | AG | 1715.91 | 188.32 | 187 | GG | 1724.26 | 196.85 | 109 | 0.61 | 0.54 | 0.28 | 0.65 | 0.58 |
| rs6658108 |  | HTR6 | GG | 1724.31 | 185.87 | 247 | AG | 1709.80 | 193.52 | 143 | AA | 1800.58 | 183.97 | 27 | 1.80 | 0.17 | 0.65 | 0.09 | 0.06 |
| rs3790757 |  | HTR6 | GG | 1727.81 | 183.18 | 307 | AG | 1714.40 | 205.48 | 110 | AA |  |  |  | 0.08 | 0.78 | 0.78 |  |  |
| rs9659997 |  | HTR6 | GG | 1736.48 | 188.73 | 263 | AG | 1708.90 | 190.68 | 135 | AA | 1664.54 | 173.41 | 19 | 2.41 | 0.09 | 0.07 | 0.13 | 0.48 |
| rs7904569 |  | HTR7 | AA | 1743.86 | 198.94 | 182 | AG | 1714.78 | 186.64 | 185 | GG | 1688.06 | 153.82 | 50 | 2.06 | 0.13 | 0.08 | 0.12 | 0.69 |
| rs4933194 |  | HTR7 | GG | 1716.94 | 179.32 | 173 | AG | 1726.86 | 193.02 | 193 | AA | 1739.34 | 208.71 | 51 | 0.06 | 0.94 | 0.97 | 0.76 | 0.74 |
| rs11596518 |  | HTR7 | AA | 1727.63 | 191.21 | 264 | AG | 1717.34 | 189.16 | 136 | GG | 1727.53 | 163.53 | 17 | 0.59 | 0.56 | 0.28 | 0.82 | 0.82 |
| rs1573935 |  | HTR7 | AA | 1719.01 | 195.90 | 115 | AG | 1726.26 | 188.77 | 209 | GG | 1726.31 | 183.36 | 93 | 0.00 | 1.00 | 0.96 | 0.92 | 0.95 |
| rs10881838 |  | HTR7 | AA | 1724.47 | 201.78 | 143 | AG | 1730.46 | 180.25 | 197 | GG | 1708.07 | 188.71 | 77 | 0.12 | 0.89 | 0.64 | 0.92 | 0.78 |
| rs12249377 |  | HTR7 | CC | 1735.40 | 196.98 | 298 | AC | 1690.54 | 154.53 | 109 | AA | 1760.23 | 259.87 | 10 | 3.87 | 0.02 | 0.06 | 0.07 | 0.02 |
| rs10785973 |  | HTR7 | CC | 1724.73 | 180.16 | 213 | AC | 1722.75 | 192.74 | 170 | AA | 1729.03 | 228.51 | 34 | 0.03 | 0.97 | 0.97 | 0.80 | 0.82 |

Note: a. Empty cells indicate that no such genotypes were found in our sample or that post hoc comparisons were not run because there were only 2 groups for this locus. Maj: Major allele; Het: Heterozygote; Min: Minor allele.

b. Results of post hoc comparisons. mh = Maj versus Het, mm = Maj versus Min, hm= Het versus Min.

c. A preliminary regression analysis was carried out to control for the influence of gender and ICV on average amygdala size. The *F*, *p* value and the *pot hoc* result in the table came from ANOVA on the residual of this regression.
